# Model Point-of-Care Ultrasound Curriculum in an Intensive Care Unit Fellowship Program and Its Impact on Patient Management

**DOI:** 10.1155/2014/934796

**Published:** 2014-11-16

**Authors:** Keith Killu, Victor Coba, Michael Mendez, Subhash Reddy, Tanja Adrzejewski, Yung Huang, Jessica Ede, Mathilda Horst

**Affiliations:** ^1^Department of Surgery/Division of Trauma and Critical Care, Henry Ford Hospital, 2799 W. Grand Boulevard, Detroit, MI 48202, USA; ^2^Department of Pulmonary and Critical Care, Henry Ford Hospital, Detroit, MI 48202, USA; ^3^Cleveland Clinic, Cleveland, OH 44195, USA; ^4^Beaumont Hospital, Royal Oaks, MI 48073, USA; ^5^Doctors Medical Center, Modesto, CA 95350, USA; ^6^Wayne State University, Detroit, MI 48202, USA

## Abstract

*Objectives*. This study was designed to assess the clinical applicability of a Point-of-Care (POC) ultrasound curriculum into an intensive care unit (ICU) fellowship program and its impact on patient care. *Methods*. A POC ultrasound curriculum for the surgical ICU (SICU) fellowship was designed and implemented in an urban, academic tertiary care center. It included 30 hours of didactics and hands-on training on models. Minimum requirement for each ICU fellow was to perform 25–50 exams on respective systems or organs for a total not less than 125 studies on ICU. The ICU fellows implemented the POC ultrasound curriculum into their daily practice in managing ICU patients, under supervision from ICU staff physicians, who were instructors in POC ultrasound. Impact on patient care including finding a new diagnosis or change in patient management was reviewed over a period of one academic year. *Results*. 873 POC ultrasound studies in 203 patients admitted to the surgical ICU were reviewed for analysis. All studies included were done through the POC ultrasound curriculum training. The most common exams performed were 379 lung/pleural exams, 239 focused echocardiography and hemodynamic exams, and 237 abdominal exams. New diagnosis was found in 65.52% of cases (95% CI 0.590, 0.720). Changes in patient management were found in 36.95% of cases (95% CI 0.303, 0.435). *Conclusions*. Implementation of POC ultrasound in the ICU with a structured fellowship curriculum was associated with an increase in new diagnosis in about 2/3 and change in management in over 1/3 of ICU patients studied.

## 1. Introduction

Ultrasound has been used since the 1940s to aid in patient management and radiologists have long appreciated its benefits. POC ultrasound is defined as ultrasonography brought to the patient and performed by the provider in real time [1]. The emergence of POC ultrasound was over 20 years ago and the initial advocates were tertiary centers applying this technology to assist the clinicians in patient management [2, 3]. Given the advantages of availability and the reproducible results, POC ultrasound became a useful tool in the clinician armamentarium for managing their patients.

POC ultrasound is becoming an integral part of many ICUs, serving as a bedside tool to assist the ICU physician in patient management. In the last two decades a more focused approach to bedside ultrasound has emerged and its use has expanded in assessing trauma patients [2, 3], hemodynamics [4–6], and disaster incidences [7, 8] as well as, many applications by the Emergency Medicine (EM) physicians across the globe [9–11], applications for procedures [10], and even found its applications in space [12, 13].

Teaching nonradiology residents, fellows, and clinicians about the use and implementation of POC ultrasound has been integrated in many academic and nonacademic centers and is currently being taught as part of the curriculum in medical schools [14, 15], in residency programs [16, 17], and through remote guidance to rural areas [18, 19] to individuals who are naïve to the technology.

Currently, POC ultrasound applications and training in the ICU [17, 20] are expanding rapidly, yet, only limited model curriculums exist for teaching and implementing POC ultrasound in the ICU, and their impact on patient care is not clear [21], and currently ultrasound training is highly recommended but not mandated by the Accreditation Council for Graduate Medical Education (ACGME) for critical care training. Adequate teaching and competency are needed to ensure patient safety; otherwise consequences of applying inadequate knowledge [22] are unknown. The Society of Critical Care Medicine and the American College of Chest Physicians offer courses for training and certification of completion of courses as well as certification of training.

Most studies have focused on the aspects of a steep learning curve [23, 24], organ or system applications, and the nonradiologist performance of specific ultrasound examination as the Focused Assessment with Sonography for Trauma (FAST) [2, 3], echocardiography [23, 24], lung exams [25], and others, as well as expanding literature emphasizing the benefits of using bedside ultrasound during procedures [10, 26, 27] and hemodynamic support [28].

The gaps in formal training programs have been identified by the American College of Chest Physicians [29] and The American Society of Echocardiography and American College of Emergency Physicians addressed in separate published consensus statements and reviews [30].

## 2. Materials and Methods

After approval by the institutional review board, a retrospective review of POC ultrasound studies and patients charts was conducted. A POC ultrasound curriculum for the surgical ICU (SICU) fellowship was designed and implemented in an urban, academic tertiary care center. As many other tertiary educational centers, we started implementing POC ultrasound in our surgical ICU where the focus was to aid in patient management and in the process and teach our surgical ICU fellows the applications of POCUS. During this process we realized the need for developing a structured curriculum for teaching. A needs assessment evaluation for a structured POC ultrasound curriculum was conducted through interviews and questionnaires to our ICU faculty and fellows, as well as conducting literature searches [16–21, 23–25]. The POC ultrasound curriculum developed in our ICU was adopted from the EM model [30] with modifications to suit the needs of patients and treating physicians in the ICU settings.

After the initial training period of the ICU fellows, they started implementing POCUS in their daily practice and management of their patients. All ultrasound exams performed were documented and reviewed by an ICU attending who is experienced in POC ultrasound, and certified by the American Registry for Diagnostic Medical Sonography (ARDMS). Data was stored as part of the patient profile and management and also as a record to evaluate the fellow's performances.

After approval by the institutional review board, a retrospective review of POC ultrasound studies and patients charts was conducted. POC ultrasound was performed by the ICU fellows and was part of the patient management and the standard of care in many institutions.

### 2.1. The Curriculum

The curriculum model was adopted from the American College of Emergency Physicians/American Society of Echocardiography (ACEP/ASE) model [30] with modifications. There is a great deal of overlap between the EM and ICU regarding the use of POC ultrasound, and our goal was to create a curriculum that is similar to a successful model which has been in place for a period of time with modifications and adjustments that are suitable for the critical care settings. The elements of the curriculum were divided into system or organ based sessions. The curriculum elements included mainly the following:

1. physics and knobology;
2. lung and pleural ultrasonography;
3. abdominal ultrasonography including gallbladder, liver, spleen, kidneys, aorta, FAST, and E-FAST;
4. vascular procedures: guidance for vascular access;
5. basic critical care echocardiography (CCE);
6. hemodynamic assessment including limited echocardiography, lungs, inferior vena cava, and internal jugular vein.

The modes of instructions included session format of 30 minutes of didactics followed by 60 minutes of hands-on sessions on models to reinforce the techniques. A total of 12 sessions were conducted every year at the beginning of the academic year for the new fellows. The fellows were required to review the ultrasound topic for the organ or system or the protocol to be discussed by referring to a handbook, textbook, and articles on POC ultrasound. The teaching sessions were provided by ICU and EM attending physicians, cardiologists, radiologists, and cardiac technicians.

Emphasis was placed on certain aspects of POC ultrasound. These aspects were as follows:

1. resuscitative: use of POC ultrasound in acute resuscitation;
2. diagnostic: use of POC ultrasound as an aid in diagnosis;
3. symptom or sign-based: use of POC ultrasound in an algorithm or protocol adopted from literature;
4. procedure guidance: use of POC ultrasound as an aid to guide a procedure;
5. therapeutic and monitoring: use of POC ultrasound in therapeutics or in physiological monitoring.

Minimum requirement for each ICU fellow was to perform 25–50 exams on respective systems or organs for a total not less than 125 exams on ICU patients during their fellowship. The requirements for achieving competency in different elements are listed in Table 1. The competency requirements were adopted from the EM curriculum [30] that has been in use for many years. Part of the total exams required by each fellow was directly supervised by the attending ICU physician before the fellow is deemed able to perform POCUS and submit reports for review. This decision was made taking into consideration other societies recommendation [30] and after direct observation of the studies performed by the ICU fellows.

Some applications, like the procedural vascular access, require fewer cases, given the prior knowledge and clinical experience with the landmark guided techniques. Documentation was done for all POC ultrasound exams and stored initially in the ultrasound unit, or on a worksheet, and some were transferred to a server system (Qpath) (Telexy Healthcare, Everett, WA, USA) that was housed in our institution where the exam video clips, still images, and reports could be stored.

Initially, the exams were supervised by one of the instructors (a total of 3 instructors performed the supervision) as shown in Table 1, after which the fellows (total of 3 fellows for the academic year) were deemed able to perform the exams on their own but still needed review by the attending physician for the final diagnosis to be confirmed.

Competency evaluations and quality assurance (QA) systems were an integral part of the curriculum. The objective of the QA process is to evaluate the images for technical competence and interpretations for clinical accuracy and to provide feedback to improve physician performance. The methods for QA included the following.

- Direct supervision of the fellow performance of POC ultrasound exam by an expert ICU physician: it is an ideal form of QA and practice performance activities. Parameters evaluated included image resolution, anatomic definition, and other image quality acquisition aspects such as gain, depth, orientation, and focus. Also the fellow's competency in POCUS was evaluated in image interpretation and forming a diagnosis.
- Providing feedback to the fellow after reviewing their POC ultrasound examination: the review and feedback were done in person and included the review of video clips and reports or commenting on reports and images that were obtained and stored. Reviewers evaluated images for accuracy and technical quality and communicated the reviews to the ICU fellow.

### 2.2. Data Collection

Data was reviewed for 203 consecutive patients (a total of 873 ultrasound exams) admitted to the SICU over a one-year period where POC ultrasound was performed as part of their management. The admitting diagnosis was made by the attending ICU physician and was established on the basis of history, physical examination, laboratory, and radiological findings. POC ultrasound was performed to aid in patient diagnosis and management. All evaluations were done almost in real time by the fellows or within minutes of the exam to ensure correct diagnosis and aid in patient management. Review of the reports was done by more than one attending physician to confirm the ultrasound diagnosis, and where there was discrepancy, the reports were not included in the final analysis. The reviewing physician was not blinded to the patient diagnosis. Outcomes measured were whether POC ultrasound led to finding of at least one new diagnosis not identified without the use of POC ultrasound and whether POC ultrasound resulted in change in management of the patient (defined as any change in medications, fluids, new laboratory or radiological tests, or new procedures).

A retrospective analysis of the results was done using Statistical Product and Service Solutions software (SPSS). The statistical methodology included retrospective descriptive frequencies and percentages of the various study types that were obtained. The two outcomes of interest (new diagnosis and change in management) were recorded as yes or no based on if they occurred in at least one of the ultrasound studies completed on each patient. The resulting overall percentages and corresponding 95% confidence intervals (CI) were calculated for the two outcomes.

## 3. Results

A POC ultrasound curriculum was developed and implemented in the SICU fellowship program. Minimum requirement for each ICU fellow was to perform 25–50 exams on respective systems or organs for a total not less than 125 exams on ICU patients during their fellowship.

During a one-year academic period, 3 ICU fellows performed 873 ultrasound studies in 203 consecutive patients, and the data was included for analysis in a retrospective clinical investigation. The ultrasound exams performed for procedural purposes were not included in the analysis.

**Figure 1:**
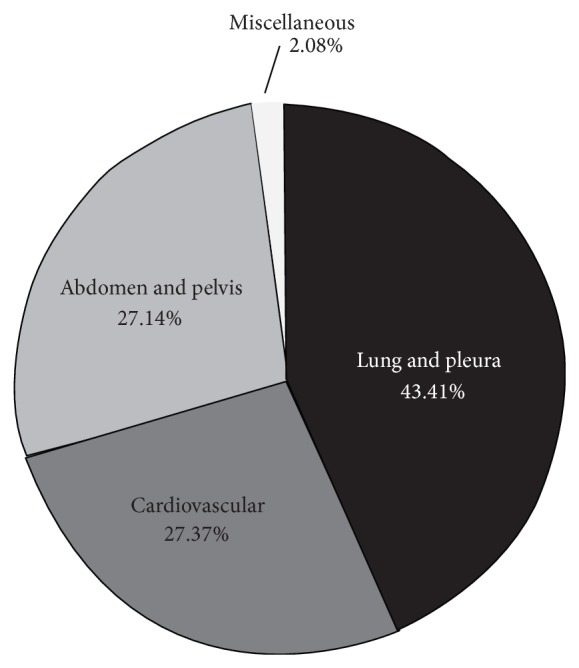
POCUS exams performed by the ICU fellows during a one-year academic period.

The most common exams performed were 379 (43.41%) lung and pleura exams, 239 cardiovascular exams (including the limited echocardiography and hemodynamic assessment) (27.37%), and 237 abdominal exams (including FAST, gallbladder, general abdomen, and pelvis) (27.14%) (Figure 1).

**Figure 2:**
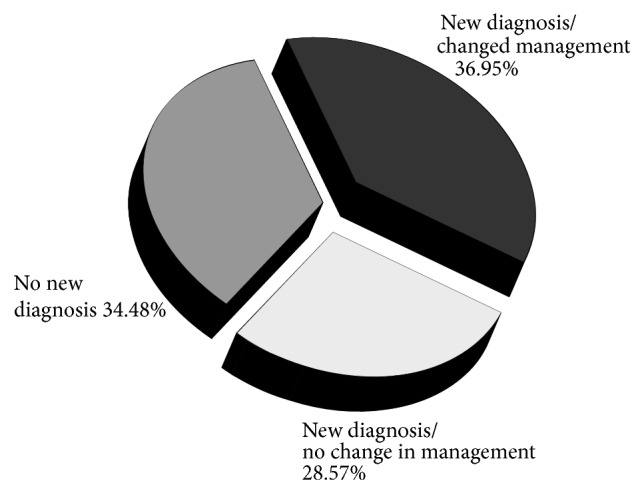
By implementing the Point-of-Care ultrasound curriculum, a new diagnosis is found in 65.52% of patients and a change in patient management was found in 36.95% of patients studied. 95% CI (0.303, 0.435).

The POC ultrasound examinations resulted in at least 1 new diagnosis in 65.52% of patients, 95% CI (0.590, 0.720), and resulted in a change in management in 36.95% of patients, 95% CI (0.303, 0.435) (Figure 2).

## 4. Discussion

POC ultrasound is currently used in the management of many disease processes seen in the critically ill patient and is considered in many instances part of the standard of care for patients management. For many years, ICU physicians have been using POC ultrasound in their practice and research of the use of POC ultrasound has been increasing. Formal curriculum is yet to be standardized for training the ICU fellows. Many societies have announced support and published statements for the use of POC ultrasound [29, 30]. The curriculum that we designed was adopted from other successful programs and after careful review of literature and years of experience. Most critical care programs that implement a curriculum use something similar in core teaching and training.

POC ultrasound research in the ICU has expanded greatly over the last 10 years. Most studies have focused on system based ultrasound exams or certain protocols [20, 23–25]. Many studies have evaluated the performance of POC ultrasound by nonradiologists in comparison to radiologists. This is the first study to report the outcomes from implementing a structured POC ultrasound curriculum in an ICU fellowship program.

Neri et al. [21] described the bedside ultrasound approach and implementing a curriculum in the ICU. Their approach was a systematic airway, breathing, circulation, deformity, and exposure (ABCDE) where all the systems were examined. The curriculum was extensive and required more time and training to achieve the required levels of competencies. Our curriculum addresses all the systems required to be examined with a focus on the presenting complaint and/or injury and critical problem-based pathways. It can serve as a more focused approach to the presenting problem, keeping in mind that at any time the operator can choose to perform a full exam as time permits. The curriculum we adopted was similar to the one endorsed by the ACEP/ASE [30] and is similar to the basic general competency (not advanced) presented by Neri.

Manno et al. [5] reported a study done in an ICU where patients were subjected to ICU-Sound Protocol. Findings showed that ultrasonographic findings modified the admitting diagnosis in 25.6%, confirmed it in 58.4%, were not effective in confirming or modifying it in 13.6%, and missed it in 2.4% of patients. Manasia et al. [23] studied the applicability of echocardiography performed by intensivists after a 10-hour tutorial sessions and found a 94% success rate in performing the exams and documented that the limited echocardiographic exams performed by the intensivists lead to a change in management in 37% of cases. Lim et al. [17] studied the development of a multidisciplinary POC ultrasound curriculum and found in the needs assessment that all ICU fellows and faculty agreed on the need for formal training and only less than 50% were comfortable with the basics of the ultrasound machines and over 90% of both groups desired more training in the echocardiography discipline.

The learning curve for POC ultrasound is steep [23, 24]. There are certain exams like the limited echocardiography exam that will require more time to master, but, generally, what we found was that implementing the curriculum with didactics and practice sessions for 30 hours followed by supervision of the trainees for a period of time, to achieve self-sufficient status, was achievable over a period of about 3 months.

Our study showed that implementing a structured POC ultrasound curriculum in the SICU fellowship program led to change in patient management with new diagnosis that would have been missed or delayed without the use of POC ultrasound. Our curriculum was designed on an existing ED model and modifications were done to adapt to our patient population. Many institutions have also adopted scanning protocols, which can be integrated in the curriculum.

The limitations of our study are that it was a single center, single ICU, retrospective data analysis with the possibility of bias being present since the POC ultrasound exam performer and interpreter were aware of the patient status and deciding the management path. Our results were not compared to the gold standard diagnostic modality for different diseases, but we relied on the opinion of expert staff members in POC ultrasound.

The curriculum we developed is not complete, and much more work and revisions will be needed. A structured curriculum needs to be conducted in the near future in a prospective manner with educational and patient outcomes measured. Those studies will help set the standard of care in POC ultrasound and help ICU physicians to use this modality in the correct and safe manner [22]. We are offering what we currently practice in our ICU in the hopes that this will stimulate more research and input from experts in the field. Following a structured curriculum with a rigorous QA process and follow-up is of utmost importance to the advancement in POC ultrasound and to our patient's care and safety [22, 31, 32].

## 5. Conclusions

Implementation of POC ultrasound in the ICU with a structured fellowship curriculum was associated with an increase in new diagnosis in about 2/3 and change in management in over 1/3 of ICU patients studied.

## Figures and Tables

**Table 1:** POC ultrasound curriculum exams requirements. To be performed by each ICU fellow during their fellowship.

Ultrasound exam element	Reviewed exams
Lung and pleura	25–50
Abdominal (including FAST)	25–50
Vascular access (and other procedures) and DVT	25–50
Basic echocardiography	25–50
Hemodynamic evaluation	25–50
